# Health-Promoting Components in Fermented Foods: An Up-to-Date Systematic Review

**DOI:** 10.3390/nu11051189

**Published:** 2019-05-27

**Authors:** Francesca Melini, Valentina Melini, Francesca Luziatelli, Anna Grazia Ficca, Maurizio Ruzzi

**Affiliations:** 1 CREA Research Centre for Food and Nutrition, Via Ardeatina 546, I-00178 Rome, Italy; francesca.melini@crea.gov.it (F.M.); valentina.melini@crea.gov.it (V.M.); 2 Department for Innovation in Biological, Agrofood and Forest systems (DIBAF), University of Tuscia, via C. de Lellis, snc, I-01100 Viterbo, Italy; f.luziatelli@unitus.it (F.L.); ficca@unitus.it (A.G.F.)

**Keywords:** fermented foods, health benefits, antioxidants, anti-hypertensives, anti-diabetics, bioactive peptides, lactose-intolerance, FODMAP, healthy diet, dietary guidelines

## Abstract

Fermented foods have long been produced according to knowledge passed down from generation to generation and with no understanding of the potential role of the microorganism(s) involved in the process. However, the scientific and technological revolution in Western countries made fermentation turn from a household to a controlled process suitable for industrial scale production systems intended for the mass marketplace. The aim of this paper is to provide an up-to-date review of the latest studies which investigated the health-promoting components forming upon fermentation of the main food matrices, in order to contribute to understanding their important role in healthy diets and relevance in national dietary recommendations worldwide. Formation of antioxidant, bioactive, anti-hypertensive, anti-diabetic, and FODMAP-reducing components in fermented foods are mainly presented and discussed. Fermentation was found to increase antioxidant activity of milks, cereals, fruit and vegetables, meat and fish. Anti-hypertensive peptides are detected in fermented milk and cereals. Changes in vitamin content are mainly observed in fermented milk and fruits. Fermented milk and fruit juice were found to have probiotic activity. Other effects such as anti-diabetic properties, FODMAP reduction, and changes in fatty acid profile are peculiar of specific food categories.

## 1. Introduction

Fermented foods are obtained through the action of microorganisms, namely bacteria, yeasts, and mycelial fungi, and their enzymes, in a process referred to as fermentation [1]. Microorganisms may be indigenously present on the substrate, or added as a starter culture, or they may be present in or on the ingredients and utensils, or in the environment. Basically, a suitable substrate, appropriate microorganism(s) and proper environmental conditions, such as temperature, pH, and moisture content must coexist in order to enable food fermentation.

During fermentation, carbohydrates and related compounds are partially oxidized and energy is released in the absence of any external electron acceptor [2]. Factors like type of sugar, limited or unlimited nutrient and oxygen availability, presence of competitive microorganisms, and time influence the process.

Fermented foods have long been produced according to knowledge passed down from generation to generation and with no understanding of the potential role of the microorganism(s) involved in the process. However, the scientific and technological revolution in Western countries made fermentation turn from a household process to a controlled one, suitable for industrial scale production systems intended for the mass marketplace.

Nowadays, indigenous fermented foods such as *dahi*, *bhalle*, *papad*, *idli*, and *dosa*, made in rural and tribal areas by using local knowledge and locally available raw materials, coexist with industrially prepared fermented food products [3]. It has been estimated that thousand different fermented foods and beverages are produced globally, using a wide range of different raw materials, microorganisms, and manufacturing techniques.

Over the last years, a renewed interest in fermented foods has been observed in Western countries largely driven by their supposed health benefits [4]. To the best of our knowledge, several reviews emphasizing health-benefits of fermented foods have been published [4,5,6,7,8,9], however none of them analyzed and discussed the healthy components that form upon fermentation of the main food matrices (namely, milk, cereals and other grains, fruit and vegetables, meat and fish) and their possible effect on human health and well-being.

The aim of this paper is thus to provide an up-to-date review of the latest studies which investigated the health-promoting components forming upon fermentation of the main food matrices, in order to contribute to understanding the functionality of different fermented food matrices and microorganisms involved in the production of healthy-components in fermented foods. Hence, the paper contributes to supporting the possible inclusion of fermented foods in national dietary recommendations worldwide.

## 2. Materials and Methods 

For the review approach, the checklist and flowchart of the PRISMA (Preferred Reporting for Systematic Reviews and Meta-Analyses) guidelines were followed [10].

### 2.1. Study Design

A preliminary SCOPUS search was performed by two authors (F.M. and V.M.), who inquired about the number of reviews on health benefits of fermented foods published over the last three years. The query string [TITLE-ABS-KEY (fermented AND foods) AND TITLE-ABS-KEY (health AND benefit) AND DOCTYPE (re) AND PUBYEAR > 2016] was used, and the preliminary screening showed that no recently published reviews addressed in a comprehensive and systematic way the analysis and discussion of the health-promoting components which form during fermentation in the main food classes (i.e., milk, cereals, fruit and vegetables, meat and fish). Based on the results obtained by the preliminary search, the aim and layout of the systematic review were designed by the authors.

A systematic literature search for peer-reviewed research papers published in the last three years (2017 to present) was thus carried out on Scopus [11], according to the terms reported in Table 1.

### 2.2. Including and Excluding Criteria, Paper Selection and Software

The research papers which were admitted for analysis were published in the last three years, so as to obtain a very up-to-date systematic review.

Duplicates, papers not accessible to the authors, and papers dealing with aspects falling beyond the scope of the paper were excluded.

Papers available in the reference list of eligible papers were also screened and selected for analysis. When they contributed to the discussion of the selected research articles, they were included in the present manuscript. 

Selected papers were collected on Microsoft Office Excel 2010 spreadsheet, and duplicates were identified.

## 3. Results and Discussion

### 3.1. Literature Search Results

**Figure 1:**
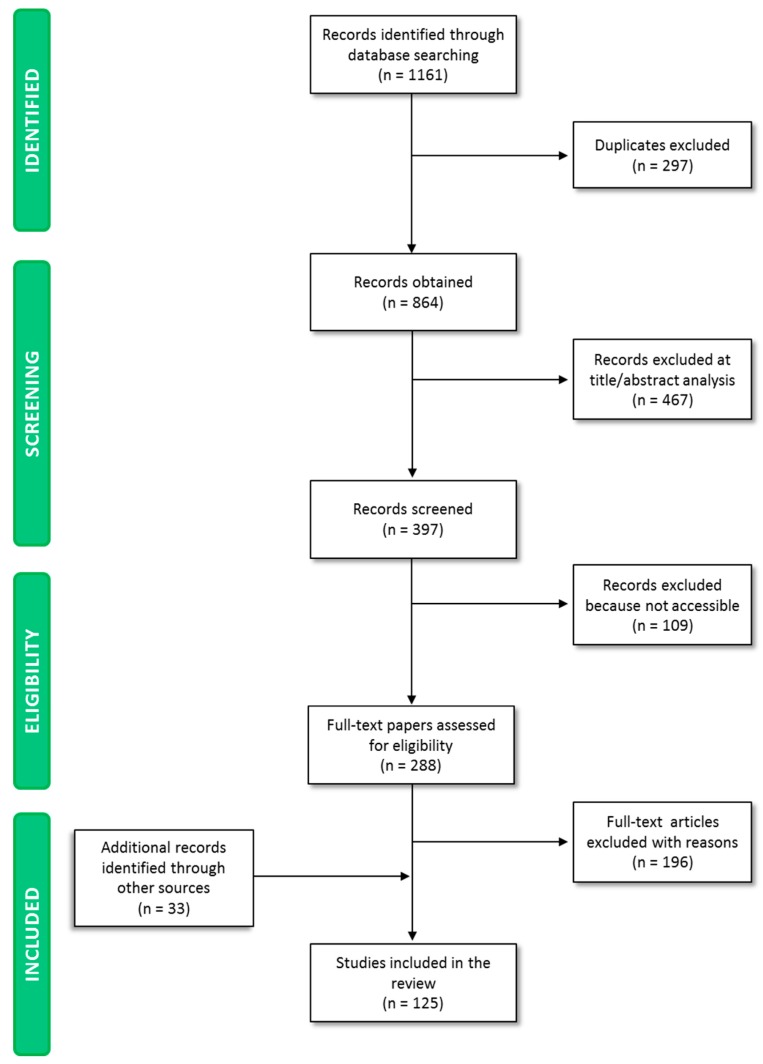
PRISMA (Preferred Reporting Items for Systematic reviews and Meta-Analyses).

The paper screening process yielded 1161 publications (Figure 1). A total of 297 papers were excluded because they were duplicates, while 467 studies were excluded on the basis of title and abstract analysis, as they dealt with aspects falling beyond the scope of the paper. A total of 109 papers were not accessible to the authors. 

This procedure resulted in 288 potentially relevant papers. A total of 196 papers were excluded on the basis of the full-text screening. Screening of reference lists of eligible papers and consultation of health/regulatory organization websites enabled the inclusion of 33 papers, which were relevant to the present analysis and discussion. A total of 125 papers were finally selected, and key information was analyzed and included in the manuscript discussion.

### 3.2. Health-Promoting Components and Activity Thereof in Fermented Foods

It has been increasingly understood that the metabolic activity of microorganisms, together with the enzymatic activities occurring in the raw material, changes the nutritive and bioactive properties of food matrices and can produce molecules with health-promoting activity [4].

In the following paragraphs, the health-promoting components developed after fermentation of the main food matrices (i.e., milk, cereals, fruit and vegetables, meat and fish) are presented and analyzed with emphasis on the starting food matrix composition and the contribution by microorganisms to the provision of additional properties beyond basic nutrition.

#### 3.2.1. Fermented Milks

Fermented milks are obtained through fermentation of milk by specific microbial consortia in which Lactic Acid Bacteria (LAB), Bifidobacteria, and yeasts grow in a protocooperative relationship. Among the microorganisms involved in the fermentation process, LAB, which comprise several members of *Lactobacillus*, *Lactococcus*, *Streptococcus*, *Leuconostoc*, and *Pediococcus* genera, are present in significant numbers and play a role in providing fermented milk with peculiar flavour, texture, and nutritional value.

Microbial starter cultures have an impact on the texture and flavour of the fermented milk, but also have a crucial role in the formation of bioactive components, which especially impart antioxidant, anti-hypertensive, anti-diabetic, and anti-allergic potential to the raw material (Table 2) [4,5,12,13].

##### Antioxidant Compounds

Food antioxidant activity is considered a crucial property of food [5], as it exerts in the human body a protective action against oxidative damage which is involved in the onset of most age- and diet-related chronic diseases [5]. Oxidative damage is caused by free radicals which are by-products of physiological reactions within human body, such as generation of calories, degradation of lipids, catecholamine response under stress, and inflammatory processes [33]. Human body can protect itself from oxidative damage through enzymatic systems, such as superoxide dismutase, glutathione peroxidase, and catalase, and non-enzymatic antioxidants comprising tocopherols, vitamin C, phenolic compounds and carotenoids, among others [33]. Antioxidant dietary supplements may help in protecting human health, however, following the increasing concerns about artificial antioxidant consumption, natural sources of antioxidants, such as fermented foods, have received greater and greater attention [34,35].

As reported by Fardet and Rock [5], dairy products have in vitro antioxidant capacity. Yoghurt and fermented milks have a higher antioxidant activity than milk. In fermented milks, it is due to the release of bioactive peptides following the proteolysis of milk proteins, especially α-lactalbumin, β-lactoglobulin, and α-casein [5,6,15,16,17].

Factors such as milk origin, milk fat content and fermenting microorganism strains can affect the antioxidant activity of fermented milks.

As far as the effect of milk origin is concerned, yogurt obtained by fermenting goat milk with *Pediococcus pentosaceus* has been found to have a higher scavenging activity than yoghurt from goat, cow and camel milk [5]. Moreover, yoghurt produced with camel milk by fermentation with *Lactobacillus rhamnosus* strain PTCC 1637 has a higher antioxidant activity than cow milk, because of the higher proline content in camel milk caseins [5]. The presence and position of the amino acids tryptophan, tyrosine, and methionine in the peptides are claimed responsible for the antioxidant activity of fermented milks as well [5].

Milk fat content can also influence yoghurt antioxidant activity, which is higher in fat free yogurt than in semi- and full-fat yogurts [15].

One more factor affecting the antioxidant activity of fermented milks is the microorganism strain responsible for the fermentation. Lim et al. [14], for instance, observed that yoghurt produced with *Lactobacillus acidophilus* strain PC16 has a higher antioxidant activity than yoghurt obtained with *Lactobacillus casei* strain PC05. Tavakoli et al. [15] compared autochthonous and commercial starter cultures of *Lb. acidophilus* and found that the type of starter culture had a significant effect (*p* < 0.05) both on proteolysis and antioxidant activity of the deriving fermented food.

Some species of LAB, such as *Lactobacillus*, *Streptococcus*, *Leuconostoc* and *Lactococcus* genera, commonly give fermented milks with high antioxidant activity [5,20]. Milk fermented with *Lb. casei* strain PRA205 has a higher radical-scavenging activity than milk fermented with *Lb. rhamnosus* [16]. Similarly to Solieri et al. [16], Ramesh et al. [36] demonstrated, when screening 19 selected *Lactobacillus* strains belonging to 10 different species for their proteolytic activity, that the production of antioxidative peptide is strain specific. *Lactobacillus plantarum* is rarely found in raw milk, however strains with probiotic properties have been isolated from camel milk, and cow or ewe raw milk cheese and whey, and the potential use of these strains to produce fermented milk beverages with enhanced health benefits has been extensively studied [37].

The antioxidant activity of fermented milk might be also increased by the formation of Conjugated Linoleic Acid (CLA), one of the major antioxidants in milk fat alongside vitamins A and E, β-carotene and coenzyme Q10 [38]. Widodo et al. [18] showed, by analyzing the presence of CLA in fermented and non-fermented milk by gas chromatography-mass spectrometry, that the formation of this class of compounds in milk is dependent on the fermentation process and on the use of selected starters, such as *Lb. casei* strain AG.

Folates have, among others, antioxidant properties which protect human body against free radical damage [39]. It has been found that LAB species are able to accumulate folate in milk, hence they have interesting potential to be used as functional cultures in fermented dairy products to replace the artificial fortification with synthetic folic acid [40]. This ability depends on species, strain and cultivation conditions [19]. For instance, *Streptococcus thermophilus*, which is one of the most important industrial dairy starters able to synthetize folates, is considered a moderate producer [40]. Tidona et al. have investigated the suitability of *Lactococcus hircilactis* and *Lactococcus laudensis* to be used as starter cultures for production of fermented milk with significant antioxidant activity and found that *Lc. hircilactis* produces moderate amounts of folates [19].

It has also been observed that the antioxidant activity and quality of fermented milk can be increased by milk ultrasound treatment before fermentation. In detail, Gholamhosseinpour and Hashemi [20] investigated the effect of ultrasound treatment on growth, carbohydrate metabolism and antioxidant activity of the probiotic strain *Lb. plantarum* strain AF1, and found that ultrasound pre-treatment of milk increases antioxidant activity because ultrasonication determines an increase in lactose hydrolysis, which implies a higher content of sugars available for the growth of LAB. Moreover, it increases the propagation ability of viable cells and the release of antioxidant components.

Milk origin and starter types have been found to affect also antioxidant activity of kefir, a fermented milk drink prepared by inoculating cow, goat or sheep milk with kefir grains which are a combination of LAB and yeasts in a matrix of proteins, lipids, and sugars. Yilmaz-Ersan et al. [41] evaluated the antioxidant activity of cow and ewe milk kefir started by either grains or commercial starters of pure or mixed-strain cultures by three different assays: 2,2′-azino-bis-3-ethylbenzothiazoline-6-sulfonic acid (ABTS) radical scavenging, 2,2-diphenyl-1-picrylhydrazyl (DPPH) radical scavenging and Ferric Reducing Antioxidant Potential (FRAP). They found that kefir made from ewe milk had higher antioxidant levels than that from cow milk. Moreover, antioxidant activity by ABTS assay was higher during fermentation of kefir by commercial strain than grains in both cow and ewe milk.

Therefore, it is felt that fermented milks can act as a source of antioxidants alternative to synthetic dietary supplements and greater attention is necessary to explore and bioprospect antioxidants from fermented foods.

##### Anti-Hypertensive Components

Intervention studies have shown that fermented milks can have a blood pressure-lowering effect [42]. The activity is exerted because anti-hypertensive peptides form from milk proteins through enzymatic breakdown by digestive enzymes or by the proteinases produced by *lactobacilli* during fermentation. The antihypertensive potential is related to the inhibition of Angiotensin-Converting Enzyme (ACE), which plays a crucial role in the regulation of blood pressure through the Renin-Angiotensin System (RAS).

ACE inhibitory peptides were identified in milks fermented with *L*. *lactis* strain NRRL B-50571 and the anti-hypertensive effect thereof were investigated in spontaneously hypertensive rats or in pre-hypertensive subjects [21,22,43,44]

Nejati et al. [23] investigated the ability of seven strains of LAB to release ACE-inhibitory peptides and synthesize GABA, and found that the milk fermented with *L. lactis* DIBCA2 had the highest ACE-inhibitory activity, whereas *L. plantarum* PU11 was identified as the most performing producer of γ-Aminobutyric acid (GABA).

Chen et al. have recently worked at the optimization of the cultural conditions to produce functional yogurt rich in GABA [24] by using *Streptococcus salivarius* subsp. *thermophilus* strain fmb5. Results showed that GABA yield is mainly affected by culture temperature, monosodium glutamate concentration and culture time. They also observed that GABA concentration, viable bacteria number and water-holding capacity of the newly formulated yoghurt was stable throughout the whole storage time [24].

##### Vitamin Content

Most vitamins cannot be synthesized by the human organism or can be only in inadequate amounts, hence an adequate dietary intake of vitamins is crucial to avoid deficiency thereof. Food processing and cooking destroy some of the vitamins normally present in raw materials, and since the diet of population is more and more made of processed foods, alternative strategies need to be adopted to assure an adequate intake of vitamins through the diet. Increasing the diversity of foods consumed, fortification of foods and supplementation are listed by the World Health Organization (WHO) in the guidelines on food fortification with micronutrients.

Some doubts have been raised about the safety of supplementing food with chemically synthesized folic acid [45,46]. Fermentation is indeed a process which allows an increase of content of some vitamins in food. For instance, over the last decades microbial fermentation has been increasingly investigated as a valuable alternative for natural folate (vitamin B_9_) production, and as a sustainable technology based on renewable resources [25].

The use of folate-producing LABs has been considered an interesting approach to bio-fortification of dairy products and fermented foods [1,25,26]. Some LAB and Bifidobacteria species are able to produce folates (vitamin B_9_) in fermented milk. Wouters et al. [27] report that when milk is processed into yogurt, the amount of folate (vitamin B_9_) may be increased to values above 200 µg/L depending on the starter cultures used and the storage condition, which can contribute to satisfying the recommended dietary allowance (400 μg DFE) [25].

As regards riboflavin (vitamin B_2_) content in fermented foods, it has been observed that its content is affected by both processing technology and the microorganisms utilized for fermentation. While some yogurt starter cultures decrease the level of riboflavin (vitamin B_2_), others significantly increase it, compared to unfermented milk [47].

Products, containing mesophilic LAB species and especially *Lactococcus* spp. as starter cultures, have also a high vitamin K content [48]. This is of paramount importance, as observational studies reported favorable associations between menaquinones (MK) intake and bone and cardiovascular health. MK-producing bacteria have been identified and selected in order to enhance the MK content of dairy products by fermentation [49]. Fu et al. [28] highlighted that the large diversity of vitamin K forms among dairy products may be related to the microbial species used in the production of fermented dairy products. MK are especially synthesized by LAB including a large number of cocci and bacilli, such as species of the genera *Carnobacterium*, *Enterococcus*, *Lactobacillus*, *Lactococcus*, *Leuconostoc*, *Oenococcus*, *Pediococcus*, *Streptococcus*, *Tetragenococcus*, *Vagococcus*, and *Weissella* [28].

Dairy products are an important source of Vitamin B_12_, which is necessary for the maintenance of the nervous system and the formation of blood cells. Its content can be increased up to 10 folds by fermentation [50].

##### Improved Protein Digestibility

During fermentation, proteolysis by microorganism enzymes can occur. Hence, a higher content of peptides and free amino acids is found in fermented foods. Gosh et al. [51] observed that free cystine, histidine, and asparagine are available in fermented cow and soy milk curd. Lorusso et al. [29] found an improved protein digestibility in yoghurt-like products obtained by fermentation with *Lb. rhamnosus* SP1, *Weissella confusa* DSM 20194, and *Lb. plantarum* T6B10.

##### Alleviation of Lactose Intolerance

It is well known that the consumption of lactose by subjects lacking adequate levels of lactase in the small intestine may result in diarrhea, bloating, abdominal pain and flatulence [52]. Hence, milk is usually avoided by lactase-deficient individuals. In contrast, fermented dairy foods, such as yoghurt, can be consumed with fewer or no symptoms, since LAB used to make yoghurt produce lactase that can achieve lactose degradation and thereby reduce symptoms [8].

Perna et al. [30] investigated the functional properties of a probiotic yogurt produced with donkey milk and *Lb. acidophilus* and *Lb. casei*. They found that the experimental yogurt had a lower lactose content than standard yoghurt.

##### Probiotic Activity

In addition to a biogenic effect, the health benefits of fermented foods may be due to a probiotic activity, that is, to the interactions of ingested live microorganisms with the host [1]. Live microorganisms must be administered in adequate amounts to confer a health benefit on the host [53].

Fermented milks and yoghurt represent indeed one of the most attractive food matrices to deliver beneficial live bacteria, as they enable the growth and/or survival of LAB, mainly including *Lactobacillus* spp. and *S. thermophilus* [54]. The efficacy is related to metabolically active cells in the gastrointestinal trait (GIT) which produce effector molecules, such as short chain fatty acids and butyrate. The challenge is to deliver viable cells to the GIT and have cell metabolic pathways genetically expressed. *Lactobacillus reuteri* was used as a probiotic microorganism by Champagne et al. [31] who used fermented milk as a carrier. Oat-based dairy fermented beverages have been also formulated with symbiotic effect of probiotic starter cultures and the prebiotic fiber β–glucan [32].

#### 3.2.2. Fermented Grain-Based Products

Cereal grains are claimed to be one of the most important sources of carbohydrates, proteins, dietary fibre (DF), vitamins, and minerals. However, their nutritional quality is inferior or poorer than other food matrices like milk and dairy products, due to i) a lower content of proteins and biological value thereof, ii) deficiency of certain essential amino acids, such as lysine, and iii) presence of determined anti-nutrients (e.g., phytic acid, tannins, and polyphenols). Fermentation is indeed the processing technology of choice to improve the nutritional properties of cereals.

Bread is the most popular cereal-based fermented food, however many other indigenous grain-based fermented foods are prepared worldwide: *idli*, *dosa*, *kishk*, *ogi*, *kenkey*, and others [55]. Alcoholic and non-alcoholic beverages are also obtained from cereal fermentation: beer, *mahewu (amahewu)*, *boza*, and *chichi* [56].

Mixed cultures of yeasts, bacteria and fungi may participate in parallel, or act in a sequential manner with a changing dominant flora during the fermentation process [56]. *Leuconostoc*, *Lactobacillus*, *Streptococcus*, *Pediococcus*, *Micrococcus* and *Bacillus* are the most common fermenting LAB species; *Aspergillus*, *Paecilomyces*, *Cladosporium*, *Fusarium*, *Penicillium,* and *Trichothecium* are the most frequently found fungi genera; whereas the most common fermenting yeasts are *Saccharomyces* species, which result in alcoholic fermentation.

Health-promoting components with antioxidant, anti-hypertensive, anti-diabetic and FODMAP-reducing activity may be found in fermented grain-based products either as a result of pre-treatment of raw materials by fermentation or by applying fermentation to the whole food system production (Table 3).

##### Antioxidant Compounds

The effect of fermentation on antioxidant activity of grains and fermented grain-based foods has been extensively investigated. Generally speaking, fermentation increases fermented food antioxidant activity by production of different compounds, depending on the raw material, the fermenting agent and the process conditions.

Different grains have been studied, from the cereal wheat to the pseudo-cereals quinoa and buckwheat, as well as legumes, such as lupin or lentil. The effect of different types of fermentation, from sourdough to yeast, has been explored. Among the components providing fermented grain-based foods with a high antioxidant activity, there are phenolic compounds, GABA, and bioactive peptides.

Fermentation enables to increase total phenolic compound content thanks to the bio-conversion of phenolic compounds from their conjugated forms to their free forms. The hydrolytic activity of enzymes produced by fermenting microorganisms promotes the structural breakdown of grain cell walls thus resulting in a greater bio-accessibility and bio-availability of bound and conjugated phenolic compounds [79]. Nevertheless, the use of analytical methods selective to phenolic compounds should be encouraged in studies on the effect of fermentation on phenolic content, since non-phenolic molecules able to react with the Folin-Ciocalteu reagent may result from fermentation, thus leading to an overestimation of phenolic content in fermented products [79].

An increase in total phenolic content has been observed in quinoa and buckwheat fermented with *P. pentosaceus* and *Lactobacillus paracasei* [57], in wheat germ, barley, rye, and buckwheat treated by fermentation with *Lb. rhamnosus* and *S. cerevisiae* [58], and in whole grain barley and oat groats fermented by LAB [80]. In detail, Rocchetti et al. [57] and Đorđević et al. [58] observed that for some raw materials the choice of the fermenting agent can modulate the content of antioxidant compounds. When quinoa is fermented with *Lb. paracasei*, the antioxidant activity is higher than when fermented by *P. pentosaceus*, in contrast to buckwheat whose fermentation with the aforesaid strains showed no difference [57]. Lorusso et al. [59] showed that the use of quinoa fermented with *Lb. plantarum* strain T6B10 and *Lactobacillus rossiae* strain T0B10 exhibited a two-fold higher antioxidant activity and total phenolic content [59] than the control.

According to Hole et al. [80], the increase in the phenolic compound content was promoted by enzymes produced by microorganisms which breakdown the cell wall matrix resulting in a greater accessibility of bound and conjugated phenolic compounds. In particular, they observed that ferulic acid content in barley and oat was respectively 81.9% and 49.9% higher than in non-fermented substrates after fermentation.

Despite the increase of total phenolic compound content is mainly considered a beneficial effect of grain processing, due to a higher antioxidant activity, it should be considered that tannins released from condensed tannins upon fermentation may bind minerals such as calcium, phosphorous, and iron [81]. However, this effect is counteracted by the degradation of oxalates and phytates that commonly reduce the bioavailability of minerals by complexing with them [81].

The metabolisation of phenolic acids by LAB was investigated by Ripari et al. who found that the release of bound ferulic acid and conversion of the obtained free ferulic acid to dihydroferulic acid and volatile metabolites was achieved by co-fermentation of *Lc. hammesii* and *Lb. plantarum*. Hence, bread quality could be improved by targeted conversion of phenolic acids during sourdough fermentation.

The increase of ferulic acid bioaccessibility after fermentation was also observed by Villalva et al. [60] in bread supplemented with bioprocessed bran. One more example of efficacy of the raw material pre-treatment by fermentation on antioxidant compound content is reported by Rashid et al. [61] who found an increased concentration of ferulic acid, organic acids, γ-oryzanol and α-tocopherol in rice bran. The healthy effect of rice bran fermentation was tested in vitro by Ryan et al. [62] who observed that a rice bran fermented by *Saccharomyces boulardii* was effective in reducing the growth of human lymphomas.

As regards the increase in GABA and peptide content after fermentation, Peñas et al. [63] showed that bread prepared with wheat sourdough produced by *Lb. brevis* CECT 8183 and a commercial protease had a higher total antioxidant activity, due to GABA and small peptide (<3 kDa) content. Curiel et al. [64] found that the application of sourdough started with *Lb. brevis* strain AM7 and *Lb. plantarum* strain C48 to legume flours allowed to obtain gluten-free bread high in GABA content. GABA content is high also when germination and sourdough fermentation of wheat, barley, chickpea, lentil and quinoa flours by strains of *Lb. plantarum*, *Lb. rossiae* and *Lactobacillus sanfranciscensis* are used [73].

Rodríguez et al. [65] investigated GABA-producing LAB strains during spontaneous fermented quinoa sourdough, and found that *Lb. plantarum* strain CRL1905, *Leuconostoc mesenteroides* strain CRL1907 and *Lb. brevis* were dominant species in the consortium.

Fermentation of rye malt sourdoughs with *Lb. reuteri* can result in formation of peptides with antioxidant activity and in accumulation of ACE inhibitory peptides [66]. Galli et al. [82] identified LAB strains useful for proteolytic and peptidase activity in sourdough: *Lb. sanfranciscensis* strain B3; *Lactobacillus farciminis* strain A11, A19 and H3; *Lb. rossiae* strain Gd40; *Lb. plantarum* strain O4; *Lb. brevis* strain A7. Bautista-Exposito et al. [67] evaluated the effect of fermentation conditions (i.e., pH and time) on proteins, peptides, phenolic content and antioxidant properties of lentils fermented by *Lb. plantarum* and Savinase^®^, an alkaline serine endopeptidase, and found that the two parameters influenced both peptides and phenolic content.

##### Anti-Hypertensive Components

Rising prevalence of hypertension has increasingly pushed the food industry towards the development of innovative food products with a reduced sodium content. This applies also to bakery products, where an extensive work of product formulation improvement and application of new biotechnologies has been done.

Sourdough fermentation represents a promising biotechnology for the production of baked goods with anti-hypertensive properties [83]. It allows, in fact, to mask the decreased salt content, thanks to the production of flavoring free amino acids and other amino acid derivatives which convey tastiness to bread, and to enrich bakery products with functional anti-hypertensive compounds.

Thanks to the proteolytic activity, LAB transform cereal matrix proteins into bioactive peptides that confer anti-hypertensive properties to fermented cereals. Peñas et al. [63] studied the effect of 21% addition of whole meal wheat sourdough (produced by *Lb. brevis* CECT 8183 and protease) on ACE inhibitory compounds. They found that sourdough fermentation, in combination with reduced sodium content may represent an interesting approach for the development of innovative bread products at reduced impact on blood pressure.

##### Anti-Diabetic Properties

The antidiabetic properties of fermented foods have been demonstrated by in vitro and in vivo studies, however the complete mechanisms responsible for this activity is not known [12]. It has been supposed that phenolic compounds, antioxidants and GABA might be responsible for the anti-diabetic activities [12]. However, other changes in cereal matrix due to fermentation contribute to the antidiabetic activity.

The application of sourdough fermentation to breadmaking allows obtaining bread with a low glycemic index (GI) and reduced starch digestibility, thanks to the formation of organic acids. Based on Harvard Medical School Recommendations which rank foods with GI ≤ 55 as low GI-food, and products with GI = 56–69 as moderate GI-food and foods with GI ≥ 70 as high GI-food, sourdough bread (GI = 54) can indeed be classified as a low GI-food [84].

In detail, lactic acid deriving from the sourdough fermentation, promotes, during heat-treatment, interactions between starch and gluten, and thus reduces starch bioavailability, and consequently, the GI of baked goods. 

Lorusso et al. [59] showed that the replacement of 20% semolina with native and fermented quinoa flour had positive effects on pasta GI.

##### Vitamin Content

Changes in the vitamin content of cereals due to fermentation vary according to the process conditions and the raw material used.

Generally speaking, animal-derived products are the main source of B_12_ vitamin, and groups of population adhering to special dietary regimes, such as vegetarians and vegans, run a high risk of vitamin B_12_ deficiency. Supplements or vitamin pills thus represent a possible alternative to the intake of vitamin B_12_ through foods.

Research studies have recently shown that fermentation enables enrichment of vitamin B_12_ in plant-based foods. For example, tempeh, which is a traditional fungal fermented Indonesian product, usually made from soybeans, is of particular interest for vegans as it contains a good amount of vitamin B_12_. It has been nevertheless observed that the use of lupin as alternative substrate and a co-culture of *Propionibacterium freudenreichii* and *Rhizopus oryzae* allow producing B12-enriched lupin tempeh [68,69]. Signorini et al. [68] found that the synergistic action of *Rhizopus* and *Propionibacterium* allowed an increase in vitamin B_12_ up to 1230 ng/g dry weight. Wolkers at al. [69] found an increase of vitamin B_12_ content up to 0.97 μg/100 g. The in situ production of active vitamin B_12_ in a mixture of aqueous cereal-based matrices (malted barley flour, barley flour and wheat aleurone) with three strains of *P. freudenreichii* has also confirmed that cereal products can be naturally fortified with active B_12_ to a nutritionally relevant level [70].

As regards other vitamin groups in fermented grain-based foods, menaquinones (MK-n) were detected with a content of about 902 μg/100 g in natto [71].

Despite the high genetic potential for folate synthesis in LAB, it has been reported a low folate content in a pearl-millet fermented porridge from Burkina Faso, *ben-saalga*, and no effect by fermentation [26]. In contrast, Kariluoto et al. [85] found folate levels can be increased in oat and barley matrices by fermentation with *S. cerevisiae* ALKO743, *Candida milleri* ABM4949, *Pseudomonas* sp. ON8 and *Janthino bacterium* sp. RB4. Saubade et al. [86] also reported that folate content increases up to 7-folds during fermentation, while Laurent-Babot and Guyot [87] have highlighted the lack of research on the effect of lactic acid fermentation on vitamin content in LAB-fermented cereal foods.

##### Improved Protein Digestibility

Fermentation can improve the protein digestibility of grains other than cereals, such as pulses, by reducing the levels of non-nutritive compounds that promote protein crosslinking (e.g., phenolic and tannin compounds) and inhibit digestive enzymes (e.g., trypsin and chymotrypsin inhibitors), as well as by production of microbial proteases, which partially degrade and release some of the proteins from the matrix [72].

Despite the protein digestibility can be increased during fermentation, an overall reduction in protein quality can be observed when alteration of sulphur amino acid content occur. Çabuk et al. [72] investigated the effect of fermentation on amino acid composition and in vitro digestibility of pea protein concentrates. They observed that fermentation is a viable method for reduction of certain non-nutritive compounds in cereal matrices, but that the use of strains with high proteolytic activity that metabolize extensively sulfur amino acids, such as *Lb. plantarum* NRRL B-4496, may have a detrimental effect on the protein quality.

Montemurro et al. [73] found that combination of germination and sourdough fermentation improves in vitro protein digestibility.

##### FODMAP Reduction

Fermentation has also been shown to play a crucial role for reduction of FODMAPs, which comprise oligosaccharides (fructans and galactans), disaccharides (lactose) and monosaccharides (fructose) and polyols (sorbitol and mannitol). These small and osmotically active molecules are poorly absorbed in the small intestine and are then rapidly fermented by bacteria in the large intestine.

FODMAP ingestion induces abdominal symptoms in people suffering from irritable bowel syndrome (IBS). A number of studies have thus been undertaken and the possible applicability of yeast fermentation in reduction of fructans and other FODMAPs has thus been shown [69,70,71,72,73].

Struyf et al. extensively investigated [74,75,76] the action of *Kluyveromyces marxianus* yeast strains in degrading fructans in whole wheat bread, as well as the addition of inulinase and the optimization of breadmaking process in terms of prolonged proofing time. Among the main results, they observed a reduction of fructan levels in the final product by more than 90% when dough was fermented with an inulinase-secreting *K. marxianus* strain, with respect to the only 56% reduction achieved by *Saccharomyces cerevisiae*. They also formulated bread prepared with a co-culture of *K. marxianus* and *S. cerevisiae* to ensure a suitable production of carbon dioxide, and observed that the conditions allowed to obtain a bread low in FODMAP and a loaf volume comparable with that of the control bread [75].

Fraberger et al. [77] isolated new strains of *S. cerevisiae* and *Torulaspora delbrueckii* from Austrian traditional sourdough, and observed that they gave interesting results in terms of degradation degree, total fructan content, and gas building capacity.

Menezes et al. [78] also highlighted that LAB and yeasts are promising tools to degrade FODMAPs. Sourdough shows a greater potential than baker’s yeast for lowering FODMAP concentrations in bread. However, they pointed out the necessity to combine enzyme activities of LAB and yeasts to formulate high quality bread also suitable for special dietary requirements.

#### 3.2.3. Fermented Fruit and Vegetables

The Food Agriculture Organization of the United Nations (FAO) and WHO report a low fruit and vegetable dietary intake as one out of the 10 major risks for morbidity and death in high-income countries. Fruit and vegetable consumption is rather associated with health benefits thanks to their content in nutrients, vitamins, minerals, DF, and non-nutritive phytochemicals whose content depends on several factors such as cultivar, agricultural practices and ripening stage.

Fruit and vegetables are highly perishable food, and fermentation has been used worldwide to prepare food products or beverages with an extended shelf-life, though the tradition of fermenting fruit and vegetable products is more widespread in Asian than in Western cultures, as highlighted by the number of traditional fermented Asian food products: *sauerkraut*, *tempeh*, *kimchi*, *gundruk*, *khalpi*, and *sinki* [88]. Compared to raw fruit and vegetables, fermented products have different nutritional characteristics due to the activity of enzymes and microorganisms during the fermentation process (Table 4). 

##### Antioxidant Compounds

Generally speaking, variation of antioxidant activity of fruit and vegetables after lactic fermentation can be observed, possibly due to the release of bioactive compounds from conjugated phytochemicals, such as phenolics [99].

As regards fruit, many papers report changes in antioxidant activity and phenolic content in fruit juices after fermentation by *Lactobacillus*. In detail, Yang et al. [89] examined the antioxidant activity of a beverage containing apples, pears, and carrots after fermentation by two strains of *Lb. plantarum* and observed an increase of the antioxidant activity with a maximum after 4–8 days fermentation. Kaprasob et al. [90] investigated the changes in physicochemical qualities, antioxidant activity and volatile compounds in cashew-apple-juice fermented by *Lb. plantarum*, and found a positive correlation of the radical-scavenging activity with vitamin-C and condensed tannins but not with hydrolysable tannins. Li et al. [91] observed enhanced DPPH and ABTS radical scavenging activity in apple juice after fermentation with *Lb. plantarum* strain ATCC14917, possibly due to the increase of 5-O-caffeoylquinic acid, quercetin, and phloretin content. Mantzourani et al. [92] observed total phenolic content and antioxidant activity greater in pomegranate juice fermented with *Lb. plantarum* strain ATCC 14917 than in unfermented juice, after 24 h of fermentation and over the time span of 28 days. In contrast, fermentation of pomegranate juice by *S. cerevisiae* was found to decrease the content of polyphenols and other bioactive compounds, except flavonoids [100]. Zhang et al. [93] report that total phenolic content of *Diospyros lotus* L. fruit decreased after fermentation with *Lb. plantarum* strain B7, while the antioxidant activity increased. All these studies highlight the significant role played by *Lb. plantarum* in fruit fermentation and widespread applicability as probiotic microorganism and/or microbial starter, as confirmed by Behera et al. [37].

Sirilun et al. [94] observed an increase in total phenolic content and antioxidant activity of *Syzygium cumini* L. fruit juice after fermentation with *Lb. paracasei* strain HII01. Bujna et al. [101] observed an increase in antioxidant activity of apricot juice following fermentation by mono- and mixed cultures of probiotic *Lactobacillus* and *Bifidobacterium* strains. Cusano et al. [96] monitored polyphenol and organic acid content during fermentation of apple juice and observed an increase in malic, lactic, quinic, pyruvic, citric, succinic, and fumaric acids, with malic being the most abundant, possibly due to their release from bound phenolics.

As far as vegetables are concerned, fermentation of tomato by LAB showed that phenolic and flavonoid content decreased after 4-week fermentation, while lycopene and antioxidant activity increased [97]. In contrast, Wiczkowski et al. [98] found that fermentation reduced the bioavailability of red cabbage anthocyanins and the human plasma antioxidant capacity. Oh et al. [102] observed an increase of saponin content in red ginseng roots after fermentation.

##### Probiotic Activity

Probiotics are mainly consumed in dairy-based food products; however, plant-based fermented foods may act as non-dairy alternatives of probiotics satisfying needs and trends for lower cholesterol, lactose-free, dairy free, vegetarian, and vegan products.

Probiotic bacteria, mainly LAB and *bifidobacteria*, are widely used during the formulation of probiotic products.

Few studies have recently reported on the “probiotication” of fruit juice. Mustafa et al. [103] investigated the effect of fermentation temperature and pH on the quality of *Punica granatum* L. juice probioticated with *Lb. plantarum*, *Lb. casei*, *Lb. bulgaricus*, and *Lactobacillus salivarius*. They found that *P. granatum* L. juice cultivated with *Lb. casei* had a better growth profile with a higher biomass density at 37 °C around pH 3.5–4.0. In addition, probiotication maintained the juice radical scavenging activity.

##### Vitamin Content

B-group vitamins are involved in several essential functions of human body such as synthesis of nucleic acids, cell metabolisms, and antioxidant activities [47]. Despite that, humans are not able to synthetize them, and dietary intake and gut microbiota are the main sources.

Plant-based fermented foods contribute to the dietary intake of B-group vitamins. Recently, Kaprasob et al. [90] have studied the effect of fermentation with 5 probiotic strains (*Lb. acidophilus*, *Lb. casei*, *Lb. plantarum*, *Lb. mesenteroides* and *B. longum*) on cashew apple juice (CAJ) B-group vitamin content and found that thiamine B_1_ content in fermented CAJ decreased significantly during fermentation, except when *Lb. acidophilus* was used as fermenting agent. *Lb. acidophilus* can, in fact, synthesize vitamin B1 and does not need it for its growth. Riboflavin (vitamin B_2_) content in fermented CAJ with 5 probiotics did not differ significantly (*p* ≥ 0.05) from non-fermented juices, while nicotinamide and vitamin B_3_ content tended to increase.

Phylloquinone (PK) is also present in fermented food products, with a detected content of about 42 μg/100 g in kimchi [71].

##### Protein Content

Shukla et al. [95] have recently investigated the fermentation process of lotus (*Nelumbo nucifera* Gaertn.) root, which has been used as an edible vegetable in East Asia for thousands of years. They found that a longer fermentation time allowed obtaining an increased total protein content (from 8.27 ± 0.86 to 392.33 ± 7.19 μg/mL).

#### 3.2.4. Fermented Meat and Fish

Dry-fermented sausages are a popular component of the diet of several people in Western countries. However, dietary guidelines worldwide recommended people to reduce meat consumption. Demand for healthier meat products with reduced fat and cholesterol content, improved fatty acid profile and added health-promoting ingredients has thus increased.

In particular, studies have been conducted to incorporate bioactive compounds in processed meat [7]. Efforts have been especially made to improve composition of fermented meat-based products.

##### Antioxidant Compounds 

Fermentation, together with drying, curing and ripening, are particular processes for meat flavour development. Fermentation also plays an important role for releasing of bioactive peptides, which have been demonstrated to have antioxidative activity (Table 5) [104].

Kęska and Stadnik [105] studied the stability of antiradical activity of protein extracts from LAB-inoculated dry-cured pork loins during long-term aging. They also evaluated their hydrolysates after simulated gastrointestinal digestion, and observed that the degradation of pork muscle proteins during gastrointestinal digestion may give rise to a wide range of peptides with antiradical properties.

Song et al. [106] evaluated the possibility of encapsulating probiotic *Bifidobacterium longum* for production of functional fermented sausages. The encapsulated *B. longum* could survive after 4 days fermentation and the products inoculated with it presented the lowest lipid oxidation level, a higher total unsaturated fatty acid content and a more desirable *n*-6/*n*-3 fatty acids than the non-inoculated control. The encapsulated *B. longum* could thus be used as a functional ingredient for production of healthier fermented meat products.

##### Fatty Acid Profile

As regards meat, the effect of addition of different fibres (e.g., citrus fibre, arabinogalactan, and inulin), of a probiotic (i.e., *Lb. rhamnosus*) and of an herbal extract to salami formulation has been investigated [107]. It was observed that their addition exerted an increase in short-chain fatty acids (SCFAs) [107].

Fermentation has been also traditionally used to improve the nutritional quality, flavour and shelf life of perishable fish. Xu et al. [108] have recently investigated the roles of three strains isolated from traditional fermented fish in FFAs liberation and lipid oxidation. *Staphylococcus xylosus* strain 135 showed the highest liberation of FFAs, most of which were polyunsaturated fatty acids (PUFA). The highest amount of PUFA oxidation in FFAs was observed with inoculation of *S. xylosus* strain 135, *Lb. plantarum* strain 120, and *S. cerevisiae* strain 31, which showed to be a promising strain for lipolysis and lipid oxidation of fermented fish.

### 3.3. Fermented Foods, Gut Microbiota and Well-Being

Adherence to healthy diet is not just providing basic nutrients to the body and limiting components of concern, such as sodium, added sugars, saturated and trans fatty acids, it is also including foods that may have a positive impact on an individual’s health well-being. The WHO defines, in fact, health as “a state of complete physical, mental and social well-being and not merely as absence of disease or infirmity” [109].

Fermented foods, as functional foods, influence human well-being thanks to a number of properties. Fermentation makes food more easily digestible, due to a pre-digestion effect. It lowers many anti-nutritional factors which inhibit digestive enzymes, such as trypsin inhibitor, and hamper an optimum absorption of minerals [81]. Fermented foods may also have a probiotic activity, hence they provide the gut with bacteria that enhances our immune function, improves digestion, and nutrient assimilation [110,111].

At its turn, gut microbiota can ferment soluble DF and β-glucans into SCFAs, such as butyrate, propionate, and acetate, which have a cholesterol-lowering effect. Several factors, namely site of fermentation, levels of fibres in the diet, gut transition time, and composition of the colonic microbiome affect the amounts of SCFAs formed during fermentation [112]. Besides contributing to cholesterol control, β-glucans have a beneficial effect on glycemic levels [113]. 

A growing body of recent research has found that the health of gut may have a direct link to the health of the brain [114,115,116], and the concept of the gut-brain axis has been also established, showing the modulatory effect of the gut microbiota composition on the brain and the central nervous system [114,117]. Intake of fermented foods can thus positively influence the gut microbiota composition, with a subsequent long-term impact on gut-brain communication.

This may be due to the fact that the gut microbiome is responsible for production of a wide array of neuro-transmitters which play a role in mental health, including dopamine, serotonin, and norepinephrine. The intake of fermented foods and probiotics and the subsequent enhancement of the gut microbiome was, in fact, found useful in reducing symptoms of depression and anxiety [114,117,118,119,120,121,122,123].

*Bifidobacterium* (e.g., *B. longum*, *B. breve*, and *B. infantis*) and *Lactobacillus* (e.g., *Lb. helveticus* , *Lb. plantarum* and *Lb. rhamnosus*) genera, which are found in several fermented foods, have shown to contribute to improving psychiatric disorder-related behaviors, among which anxiety, depression, obsessive-compulsive disorder, and memory abilities, as well as to attenuating stress responses. They can, in fact, maintain adequate levels of neuropeptide brain-derived neurotrophic factor, which are known to be low in depression. The bidirectional communication between the central nervous system (CNS) and the gut microbiota also influences the brain health and cognitive functions [118,119].

As there is still a limited number of clinical trials which link fermented foods to the role and modulation of the gut microbiota, and to the state of well-being [114,124], additional research in the field should be prompted.

### 3.4. Fermented Foods in National Dietary Guidelines

National dietary guidelines are an important part of national nutrition policies. They are recommendations, in the form of guidelines, for healthy eating and well-being formulated by scientists and health professionals on the basis of the latest scientific evidence and food consumption data [125].

In detail, they give advice on the food groups to prefer, on the amounts of foods (e.g., servings per day) to consume, and on the dietary patterns to follow. National dietary guidelines all over the world share recommendations such as a regular intake of fruit and vegetables, a preference for unsaturated fats, and for a daily intake of salt lower than 5 g. However, national dietary guidelines are country-specific and unique to the population and country that developed them. They are related and influenced by the availability of food products in the specific country, and by national dietary and cultural characteristics. Moreover, they are formulated by taking into consideration the national priorities in public health, which may differ from country to country. Dietary guidelines should also be culturally acceptable and understandable for the population.

As regards fermented foods, a few countries worldwide, such as Kenya, South Africa, Australia, India, Sri Lanka, Oman, Qatar, and Bulgaria, have so far included recommendation for consumption thereof in their national dietary guidelines. In these countries indigenous and traditional fermented foods are also available and represent a rooted element of the national culture.

At the moment, reference to fermented foods as being health-promoting is common. They contain health-promoting components, can be used as a probiotic carrier, can contribute to the diversity of gut microbiome, and indirectly impact on mental health and other disorders. The early introduction of fermented foods in the diet has been supposed to lessen children desire to over-consume sweet foods [126]. Sugar preference may be tied, in fact, to the bacteria in the gut and children get their gut microbioma from the early stage of their life.

Nonetheless there is a lack of solid scientific foundations of such claims and there is still a need for fundamental research on randomized, controlled, clinical trials to measure the quantitative repeatable effects of fermented foods in the different groups of the populations to eventually justify their inclusion on national food guidelines. Identification of sound relationship between fermented foods and health is a pre-requisite for a stepwise inclusion of this food category in population dietary habits and hence in national dietary guidelines. Moreover, chemical and microbiological composition of fermented foods should be exactly known in order to ensure the claimed effect on health and well-being.

## 4. Conclusions

During food fermentation, a number of chemical changes occur in the components of the raw matrix, which thus results in products with improved nutritional properties and healthy effects. A higher bioactive molecule content and an improved antioxidant activity were found in fermented milks, cereals, fruit and vegetables, meat and fish. Anti-hypertensive peptides were detected in fermented milk and cereals. Changes in vitamin content were mainly observed in fermented milk and fruits. Fermented milk and fruit juice were found to have probiotic activity. Other effects such as antidiabetic properties, FODMAP reduction, and changes in fatty acid profile were peculiar of specific food categories.

Fermented foods can also influence human well-being. The intake of fermented foods and probiotics and the subsequent enhancement of the gut microbiome may have a modulatory effect on the brain and the central nervous system.

The occurrence of healthy components and the activity thereof make fermented foods worthy of recommendation of regular consumption and inclusion in worldwide dietary guidelines. There is however a need for research on clinical trials to measure the effects of fermented foods in the different groups of the population.

The paper also allowed to identify which microorganism strains are more suitable for optimal production of healthy-components in the different food matrices. Hence, the review might be a baseline in engineering and designing novel fermented foods.

## Figures and Tables

**Table 1:** Query strings used for the database search on Elsevier’s SCOPUS, the largest bibliographic database of peer-reviewed literature.

Query String	No. of Documents Found
(TITLE-ABS-KEY (fermented AND food) OR TITLE-ABS-KEY (fermentation) AND TITLE-ABS-KEY (healthy AND component) OR TITLE-ABS-KEY (health AND benefit) AND TITLE-ABS-KEY (milk)) AND DOCTYPE (ar) AND PUBYEAR > 2016	58
(TITLE-ABS-KEY (fermented AND food) OR TITLE-ABS-KEY (fermentation) AND TITLE-ABS-KEY (healthy AND component) OR TITLE-ABS-KEY (health AND benefit) AND TITLE-ABS-KEY (cereals)) AND DOCTYPE (ar) AND PUBYEAR > 2016	16
(TITLE-ABS-KEY (fermented AND food) OR TITLE-ABS-KEY (fermentation) AND TITLE-ABS-KEY (healthy AND component) OR TITLE-ABS-KEY (health AND benefit) AND TITLE-ABS-KEY (pulses)) AND DOCTYPE (ar) AND PUBYEAR > 2016	1
(TITLE-ABS-KEY (fermented AND food) OR TITLE-ABS-KEY (fermentation) AND TITLE-ABS-KEY (healthy AND component) OR TITLE-ABS-KEY (health AND benefit) AND TITLE-ABS-KEY (legumes)) AND DOCTYPE (ar) AND PUBYEAR > 2016	5
(TITLE-ABS-KEY (fermented AND food) OR TITLE-ABS-KEY (fermentation) AND TITLE-ABS-KEY (healthy AND component) OR TITLE-ABS-KEY (health AND benefit) AND TITLE-ABS-KEY (grains)) AND DOCTYPE (ar) AND PUBYEAR > 2016	27
(TITLE-ABS-KEY (fermented AND food) OR TITLE-ABS-KEY (fermentation) AND TITLE-ABS-KEY (healthy AND component) OR TITLE-ABS-KEY (health AND benefit) AND TITLE-ABS-KEY (meat)) AND DOCTYPE (ar) AND PUBYEAR > 2016	11
(TITLE-ABS-KEY (fermented AND food) OR TITLE-ABS-KEY (fermentation) AND TITLE-ABS-KEY (healthy AND component) OR TITLE-ABS-KEY (health AND benefit) AND TITLE-ABS-KEY (fish)) AND DOCTYPE (ar) AND PUBYEAR > 2016	9
(TITLE-ABS-KEY (fermented AND food) OR TITLE-ABS-KEY (fermentation) AND TITLE-ABS-KEY (healthy AND component) OR TITLE-ABS-KEY (health AND benefit) AND TITLE-ABS-KEY (fruit)) AND DOCTYPE (ar) AND PUBYEAR > 2016	39
(TITLE-ABS-KEY (fermented AND food) OR TITLE-ABS-KEY (fermentation) AND TITLE-ABS-KEY (healthy AND component) OR TITLE-ABS-KEY (health AND benefit) AND TITLE-ABS-KEY (vegetable)) AND DOCTYPE (ar) AND PUBYEAR > 2016	28
(TITLE-ABS-KEY (fermented AND food) OR TITLE-ABS-KEY (fermentation) AND TITLE-ABS-KEY (antioxidants) AND TITLE-ABS-KEY (milk) OR TITLE-ABS-KEY (cereals) OR TITLE-ABS-KEY (pulses) OR TITLE-ABS-KEY (legumes) OR TITLE-ABS-KEY (grains) OR TITLE-ABS-KEY (meat) OR TITLE-ABS-KEY (fish) OR TITLE-ABS-KEY (fruit) OR TITLE-ABS-KEY (vegetable)) AND DOCTYPE (ar) AND PUBYEAR > 2016	383
(TITLE-ABS-KEY (fermented AND food) OR TITLE-ABS-KEY (fermentation) AND TITLE-ABS-KEY (anti-hypertensive) AND TITLE-ABS-KEY (milk) OR TITLE-ABS-KEY (cereals) OR TITLE-ABS-KEY (pulses) OR TITLE-ABS-KEY (legumes) OR TITLE-ABS-KEY (grains) OR TITLE-ABS-KEY (meat) OR TITLE-ABS-KEY (fish) OR TITLE-ABS-KEY (fruit) OR TITLE-ABS-KEY (vegetable)) AND DOCTYPE (ar) AND PUBYEAR > 2016	7
(TITLE-ABS-KEY (fermented AND food) OR TITLE-ABS-KEY (fermentation) AND TITLE-ABS-KEY (vitamins) AND TITLE-ABS-KEY (milk) OR TITLE-ABS-KEY (cereals) OR TITLE-ABS-KEY (pulses) OR TITLE-ABS-KEY (legumes) OR TITLE-ABS-KEY (grains) OR TITLE-ABS-KEY (meat) OR TITLE-ABS-KEY (fish) OR TITLE-ABS-KEY (fruit) OR TITLE-ABS-KEY (vegetable)) AND DOCTYPE (ar) AND PUBYEAR > 2016	117
(TITLE-ABS-KEY (fermented AND food) OR TITLE-ABS-KEY (fermentation) AND TITLE-ABS-KEY (probiotic) AND TITLE-ABS-KEY (milk) OR TITLE-ABS-KEY (cereals) OR TITLE-ABS-KEY (pulses) OR TITLE-ABS-KEY (legumes) OR TITLE-ABS-KEY (grains) OR TITLE-ABS-KEY (meat) OR TITLE-ABS-KEY (fish) OR TITLE-ABS-KEY (fruit) OR TITLE-ABS-KEY (vegetable)) AND DOCTYPE (ar) AND PUBYEAR > 2016	430
(TITLE-ABS-KEY (fermented AND food) OR TITLE-ABS-KEY (fermentation) AND TITLE-ABS-KEY (lactose intolerance) AND TITLE-ABS-KEY (milk) OR TITLE-ABS-KEY (cereals) OR TITLE-ABS-KEY (pulses) OR TITLE-ABS-KEY (legumes) OR TITLE-ABS-KEY (grains) OR TITLE-ABS-KEY (meat) OR TITLE-ABS-KEY (fish) OR TITLE-ABS-KEY (fruit) OR TITLE-ABS-KEY (vegetable)) AND DOCTYPE (ar) AND PUBYEAR > 2016	12
(TITLE-ABS-KEY (fermented AND foods) AND TITLE-ABS-KEY (well-being)) AND DOCTYPE (ar) AND PUBYEAR > 2016	18
	**1161**

**Table 2:** Health-promoting compounds in fermented milks.

Health-Promoting Activity	Health-Promoting Compounds	Raw Food Matrices	Fermenting Microorganism(s)	References
Antioxidant activity	Phenolic compounds, GABA ^1^, peptides, CLA ^2^, folates (vitamin B_9_)	Goat milk	*Pediococcus pentosaceus*	[5]
		Camel milk	*Lactobacillus rhamnosus* strain PTCC 1637	[5]
		Milk	*Lactobacillus acidophilus* strain PC16	[14]
		Milk	*Lb. acidophilus*	[15]
		Milk	*Lactobacillus casei* strain PRA205	[16]
		Skimmed milk	*Lactobacillus delbrueckii* spp. *bulgaricus* strain LB340	[17]
		Milk	*Lb. casei* strain AG	[18]
		Milk	*Lactococcus hircilactis/Lactococcus laudensis*	[19]
		Milk	*Lactobacillus plantarum* strain AF1	[20]
Anti-hypertensive activity	ACE ^3^ inhibitory peptides and GABA	Skimmed milk	*Lactococcus lactis* strain NRRL B-50571	[21,22]
		Milk	*Lactobacillus* spp.	[23]
		Milk	*Streptococcus salivarius* subsp. *thermophilus* strain fmb5	[24]
Increase of vitamin content	Folate (vitamin B_9_), vitamin K, riboflavin (vitamin B_2_)	Milk	LAB and Bifidobacteria species	[1,25,26]
		Milk processed into yogurt	-	[27]
		Milk	Species of the genera *Carnobacterium*, *Enterococcus*, *Lactobacillus*, *Lactococcus*, *Leuconostoc*, *Oenococcus*, *Pediococcus*, *Streptococcus*, *Tetragenococcus*, *Vagococcus*, and *Weissella*	[28]
Improvement of protein digestibility	-	Kefir	*Lb. rhamnosus* SP1 *Weissella confusa* DSM 20194 *Lb. plantarum*	[29]
Alleviation of lactose intolerance		Donkey milk	*Lb. acidophilus* and *Lb. casei*	[30]
Probiotic activity		Milk	*Lactobacillus reuteri*	[31]
		Oat-based dairy fermented beverages	-	[32]

^1^ γ-Aminobutyric Acid; ^2^ Conjugated Linoleic Acid; ^3^ Angiotensin-Converting Enzyme.

**Table 3:** Health-promoting compounds in fermented grain-based products.

Health-Promoting Activity	Health-Promoting Compounds	Raw Food Matrices	Fermenting Microorganism(s)	References
Antioxidant activity	Phenolic compounds, GABA ^1^, peptides, CLA ^2^, folates (vitamin B_9_)	Quinoa and buckwheat	*P. pentosaceus* and *Lactobacillus paracasei*	[57]
		Wheat germ, barley, rye and buckwheat	*Lb. rhamnosus* and *Saccharomyces cerevisiae*	[58]
		Quinoa fermented	*Lb. plantarum* strain T6B10 and *Lactobacillus rossiae* strain T0B10	[59]
		Bread supplemented with bioprocessed Bran	Yeast, xylanase enzyme and combination thereof	[60]
		Rice bran	*Pediococcus acidilactici*, *L. lactis* and *Pediococcus pentoseous*	[61]
		Rice bran	*Saccharomyces boulardii*	[62]
		Bread prepared with wheat sourdough	*Lb. brevis* CECT 8183 and a commercial protease	[63]
		Sourdough and legume flours	*Lb. brevis* strain AM7 and *Lb. plantarum* strain C48	[64]
		Spontaneous fermented quinoa sourdough	*Lb. plantarum* strain CRL1905, *Leuconostoc mesenteroides* strain CRL1907 and *Lb. brevis*	[65]
		Rye malt sourdoughs	*Lb. reuteri*	[66]
		Lentils	*Lb. plantarum* and Savinase^®^	[67]
Anti-hypertensive activity	ACE ^3^ inhibitory peptides and GABA	Whole meal wheat sourdough	*Lb. brevis* CECT 8183 and protease	[63]
				
Vitamin content	Folate (vitamin B_9_), vitamin K, riboflavin (vitamin B_2_)	Lupin - tempeh	Co-culture of *Propionibacterium freudenreichii* and *Rhizopus oryzae*	[68,69]
			*Rhizopus and Propionibacterium*	[68]
		Cereal-based matrices (malted barley flour, barley flour and wheat aleurone)	Three strains of *P. freudenreichii*	[70]
		Natto	-	[71]
Protein hydrolysis		Pea proteins	-	[72]
		Germination and sourdough fermentation	-	[73]
Anti-diabetic properties		Pasta formulated with 20% fermented quinoa flour	-	[59]
FODMAP Reduction		Whole wheat bread	*Kluyveromyces marxianus* yeast strains	[74,75,76]
		Austrian traditional sourdough	*S. cerevisiae and Torulaspora delbrueckii*	[77]
			LAB and yeasts	[78]

^1^ γ-Aminobutyric Acid; ^2^ Conjugated Linoleic Acid; ^3^ Angiotensin-Converting Enzyme.

**Table 4:** Health-promoting compounds in fermented fruit and vegetables.

Health-Promoting Activity	Health-Promoting Compounds	Raw Food Matrices	Fermenting Microorganism(s)	References
Antioxidant activity	Phenolic compounds, GABA ^1^, peptides, CLA ^2^, folates (vitamin B_9_)	Beverage containing apples, pears, and carrots	Two strains of *Lb. plantarum*	[89]
		Cashew-apple-juice	*Lb. plantarum*	[90]
		Apple juice	*Lb. plantarum* strain ATCC14917	[91]
		Pomegranate juice fermented	*Lb. plantarum* strain ATCC 14917	[92]
		*Diospyros lotus* L. fruit	*Lb. plantarum* strain B7	[93]
		*Syzygium cumini* L fruit juice	*Lb. paracasei* strain HII01	[94]
		Apricot juice	Mono- and mixed cultures of probiotic *Lactobacillus* and *Bifidobacterium* strains	[95]
		Apple juice	-	[96]
		Tomato	LAB	[97]
		Red cabbage	-	[98]
Vitamin content	Vitamin K (Phylloquinone)	Cashew apple juice	5 probiotic strains (*Lb. acidophilus*, *Lb. casei*, *Lb. plantarum*, *Lb. mesenteroides* and *B. longum*)	[90]
		Kimchi		[71]
Protein hydrolysis		Lotus (*Nelumbo nucifera* Gaertn.) root	-	[95]

^1^ γ-Aminobutyric Acid; ^2^ Conjugated Linoleic Acid.

**Table 5:** Health-promoting compounds in fermented meat and fish.

Health-Promoting Activity	Health-Promoting Compounds	Raw Food Matrices	Fermenting Microorganism(s)	References
Antioxidant activity	Phenolic compounds, GABA ^1^, peptides, CLA ^2^, folates (vitamin B_9_)	Dry-cured pork loins	Probiotic Strains of LAB	[105]
		Functional fermented sausages	Encapsulation of probiotic *Bifidobacterium longum*	[106]

^1^ γ-Aminobutyric Acid; ^2^ Conjugated Linoleic Acid.
